# Prognostic PET [^11^C]-acetate uptake is associated with hypoxia gene expression in patients with late-stage hepatocellular carcinoma – a bench to bed study

**DOI:** 10.1186/s40644-024-00685-9

**Published:** 2024-03-22

**Authors:** Keith Wan Hang Chiu, Kel Vin Tan, Xinxiang Yang, Xiaoqiang Zhu, Jingjing Shi, Chi-Leung Chiang, Lawrence Chan, Yuan Hui, Pek-Lan Khong, Kwan Man, Jason Wing Hon Wong

**Affiliations:** 1 Department of Radiology and Imaging, Queen Elizabeth Hospital, Hong Kong SAR, China; 2 Department of Oncology, MRC Oxford Institute for Radiation Oncology, University of Oxford, Oxford, UK; 3 Department of Diagnostic Radiology, School of Clinical Medicine, LKS Faculty of Medicine, The University of Hong Kong, Hong Kong SAR, China; 4 Department of Surgery, School of Clinical Medicine, LKS Faculty of Medicine, The University of Hong Kong, Hong Kong SAR, China; 5 School of Biomedical Sciences, LKS Faculty of Medicine, The University of Hong Kong, Hong Kong SAR, China; 6 Department of Clinical Oncology, School of Clinical Medicine, LKS Faculty of Medicine, The University of Hong Kong, Hong Kong SAR, China; 7 Department of Health Technology and Informatics, The Hong Kong Polytechnic University, Hong Kong SAR, China; 8 Guangdong Provincial People’s Hospital, Guangdong Academy of Medical Science, Guangzhou, China; 9 Clinical Imaging Research Center (CIRC), Yong Loo Lin School of Medicine, National University of Singapore, Singapore, Singapore

**Keywords:** Hepatocellular carcinoma, Hypoxia, PET imaging, RNA sequencing, [^11^C]-acetate

## Abstract

**Background:**

Positron Emission Tomography (PET) with combined [^18^F]-FDG and [^11^C]-acetate (dual-tracer) is used for the management of hepatocellular carcinoma (HCC) patients, although its prognostic value and underlying molecular mechanism remain poorly understood. We hypothesized that radiotracer uptake might be associated with tumor hypoxia and validated our findings in public and local human HCC cohorts.

**Methods:**

Twelve orthotopic HCC xenografts were established using MHCC97L cells in female nude mice, with 5 having undergone hepatic artery ligation (HAL) to create tumor hypoxia in vivo. Tumors in both Control and HAL-treated xenografts were imaged with [^11^C]-acetate and [^18^F]-FDG PET-MR and RNA sequencing was performed on the resected tumors. Semiquantitative analysis of PET findings was then performed, and the findings were then validated on the Cancer Genome Atlas Liver Hepatocellular Carcinoma (TCGA-LIHC) cohort and patients from our institution.

**Results:**

HAL-treated mice showed lower [^11^C]-acetate (HAL-treated vs. Control, tumor-to-liver SUV ratio (SUV_TLR_): 2.14[2.05–2.21] vs 3.11[2.75–5.43], *p* = 0.02) but not [^18^F]-FDG (HAL-treated vs. Control, SUV_TLR_: 3.73[3.12–4.35] vs 3.86[3.7–5.29], *p* = 0.83) tumor uptakes. Gene expression analysis showed the PET phenotype is associated with upregulation of hallmark hypoxia signature. The prognostic value of the hypoxia gene signature was tested on the TCGA-LIHC cohort with upregulation of hypoxia gene signature associated with poorer overall survival (OS) in late-stage (stage III and IV) HCC patients (*n* = 66, OS 2.05 vs 1.67 years, *p* = 0.046). Using a local cohort of late-stage HCC patients who underwent dual-tracer PET-CT, tumors without [^11^C]-acetate uptake are associated with poorer prognosis (*n* = 51, OS 0.25 versus 1.21 years, *p* < 0.0001) and multivariable analyses showed [^11^C]-acetate tumor uptake as an independent predictor of OS (HR 0.17 95%C 0.06–0.42, *p* < 0.0001).

**Conclusions:**

[^11^C]-acetate uptake is associated with alteration of tumor hypoxia gene expression and poorer prognosis in patients with advanced HCC.

**Supplementary Information:**

The online version contains supplementary material available at 10.1186/s40644-024-00685-9.

## Background

Hepatocellular Carcinoma (HCC) is the fourth most common cancer worldwide, accounting for nearly 850,000 cases annually [1]. With over half of the patients diagnosed at advanced tumor stage precluding them from curative surgery, locoregional and/or systemic treatment are the mainstay therapeutic options in the management of HCC [2]. However, as the efficacy of these non-surgical treatments is heavily dependent on the pathophysiological parameters of the tumor, a detailed understanding of the molecular and cellular processes of HCC pathogenesis is critical. Tissue-based assays are currently the only method that can reliably detect molecular changes in tumors [3]. As yet, histological sampling is prone to sampling error and fails to account for the inter and intra-tumoral heterogeneity of classically characterized HCC, let alone the risks of potential life-threatening complications such as hemorrhage [4]. A less invasive method that allows the detection of molecular changes and identification of HCC subtypes is urgently needed.

Despite the critical role conventional cross-sectional imaging plays in the diagnosis and management of HCC, limited radiological features are reliable biomarkers for predicting patient prognosis or reflect underlying tumor biology [5]. Over the past decade, positron emission tomography (PET) with combined [^18^F]-FDG and [^11^C]-acetate (dual-tracer) PET is being used as a research tool or as an adjunct imaging modality in some centers worldwide, although its prognostic significance and underlying molecular mechanism of the radiotracers remains inconclusive [6–9].

Given that tumor hypoxia is associated with profound changes in metabolism, the purpose of this study was to examine whether [^18^F]-FDG and [^11^C]-acetate (dual-tracer) PET may provide semi-quantitative measure of tumor hypoxia. While hypoxia is well known to be associated with [^18^F]-FDG uptake in HCC, only around 50% of HCC is [^18^F]-FDG avid, thus limiting its utility in real world prognostication. In recent year, acetate is found to be a metabolite as well as epigenetic regulator in many cancers including HCC [10]. With sensitivity of [^11^C]-acetate uptake as high as 90% in clinical practice, it has the potential to be an ideal non-invasive biomarker [6].

To do so, we created orthotopic mice model bearing human HCC cell line MHCC97L as our preclinical model for investigation, which underwent hepatic artery ligation (HAL) to accentuate hypoxic conditions. Dual-tracer PET and expression profiling were performed on the mice to identify radiological and molecular associations with tumor hypoxia. As hypoxia has been associated with poor prognosis, we further demonstrate the potential of [^11^C]-acetate PET for HCC prognostication.

This manuscript is written following STROBE checklist and ARRIVE Guidelines.

## Methods

### Subcutaneous, orthotopic HCC xenografts and PET-MR imaging

Full materials and methods for cell cultures and animal experiments are presented in the supplemental information (SI).

First, the subcutaneous tumor block was excised and subsequently cut into small tumor cube for implantation into the liver. Orthotopic tumor inoculation was performed on 14 mice [11]. To mimic the effect of hypoxia in HCC tumors in vivo, HAL was performed on the common hepatic artery (CHA) of each mouse at the time of tumor implantation [12]. This was done by tying a fine thread around the main branch of the hepatic artery under an operating microscope. Mice were randomized to receive sham-operation (*n* = 7) or HAL (*n* = 7) during tumor implantation. All mice that received a simple sham-operation survived while 2 out of 7 mice that underwent HAL developed a poor general condition and were culled and excluded from the study. In total, 5 HAL-treated mice and 7 sham-operated (Control) xenografts were used for imaging studies. Tumor growth was monitored by MRI and four weeks after injection, all mice underwent dual-tracer PET imaging on the nanoScan® PET-MRI system (Mediso Ltd., Hungary). Mice were anesthetized via inhalation of 5% isoflurane/oxygen gas mixture on a pre-warmed mouse bed. The tail of each mouse was warmed gently before intravenous injection of [^11^C]-acetate (18.5 ± 2 MBq) followed by a 30-min uptake before PET imaging. One hour after completion of [^11^C]-acetate PET, mice were re-injected with [^18^F]-FDG (7.4 ± 1 MBq) intravenously, followed by a 60-min uptake. Mice were maintained using 2% isoflurane/oxygen mixture during the uptake and imaging periods, and body temperature was maintained at 37 °C during this time. Static PET acquisitions were performed for 20 min in 1:3 coincidence and normal count mode. PET images were reconstructed using the three-dimensional Ordered Subsets Expectation Maximization (3D-OSEM) algorithm (Tera-Tomo, Mediso Ltd., Hungary), with radionuclide decay, normalization, random, scatter, attenuation, and dead time corrections applied to the data, resulting in a matrix of 0.3 mm^3^. The PET and MR images were co-registered automatically and analyzed using InterView™ FUSION v3.03 (Mediso Ltd., Hungary). For each scan, tumor volumes of interest (VOI) were delineated on MR images and recorded. The same VOI was applied manually in the PET images and presented as the standard uptake value (SUV). A 3 mm^3^ of VOI was placed on the liver, avoiding areas of visible hepatic vascular and biliary structures to provide an internal control. SUV was calculated using the following equation: SUV = C_PET_(*t*)/(ID/BW), where C_PET_(*t*) is the measured activity in VOI, ID is the injected dose measured in kilobecquerels, and BW is the mouse body weight in kilograms, assuming a tissue density of 1 g/mL. Tumor-to-liver ratio (SUV_TLR_) were measured to normalize the tumor uptake of all tracers.

### RNA sequencing and analyses

To examine the molecular changes underlying hypoxia and reduced [^11^C]-acetate uptake, tumors were excised for RNA sequencing (RNA-seq). A total of 12 RNA samples consisting of 5 HAL-treated and 7 Control mice (in RNALater) were subjected to transcriptome sequencing (see SI for information). For gene expression analysis, featureCounts [13] was used to count the number of reads aligning to genes based on UCSC canonical annotations. Differential expression analysis was performed using DESeq2 version 1.28 [14]. Gene set enrichment analysis (GSEA) [15] was performed to evaluate the enrichment of specific biological pathways.

### Analysis of TCGA cohort

Normalized RNA-seq data of 371 HCC patients along with their clinical data were retrieved from TCGA-LIHC cohort through the UCSC Xena Browser [16]. To predict if each sample corresponded to a high or low acetate uptake state, a gene signature was developed based on differentially expressed (adjusted *p* value < 0.05 & |logFC|> 1) genes between the Control and HAL-treated group. A higher logFC threshold was used to ensure the gene signature could be more easily translated to other cohorts. The Nearest-Template Prediction (NTP) algorithm [17] was used to group the TCGA samples based on the expression of this gene signature in each sample. A sample was considered to have been successfully assigned to a group if the Benjamini-Hochberg-corrected false discovery rate (FDR) was < 0.2 for the respective group prediction. To determine which NTP group is associated with high or low acetate uptake, we selected genes that are upregulated in the Control group (i.e. when acetate uptake is high) and used GSVA [18] to calculate an “acetate signature score” for each TCGA-LIHC sample to determine which NTP group is associated with high and low acetate uptake respectively. Kaplan–Meier plots were generated on all successfully assigned TCGA-LIHC patients (*n* = 295) and late stage (Stage III&IV) patients (*n* = 66) to compare the difference in prognosis based on OS.

### Analysis of local cohort

Dual-tracer PET-CT were performed as per standard protocol in our institute [19]. Consecutive patients who underwent dual-tracer PET-CT between January 2015 and December 2019 were retrospectively identified. Those with treatment naïve HCC who underwent dual-tracer PET-CT for staging purposes were included for analysis. PET-CT images were interpreted by a radiologist and a clinical oncologist by consensus, both with > 5 years of dual-tracer PET-CT using a dedicated workstation (ADW4.7, GE Healthcare). Intrahepatic HCC were interpreted both visually and semi-quantitatively. A lesion is considered positive for each radiopharmaceutical if it has increased metabolism visually over background liver parenchyma that is supported by an increase in standardized uptake value (SUV) (see SI for information). Clinical details, including patient demographics and outcomes, were obtained from electronic medical record review. Kaplan–Meier plots were generated on all HCC patients who underwent staging (*n* = 197) and those with late-stage disease (*n* = 51) to compare the difference on OS.

### Statistical analysis

Statistical analyses were performed using GraphPad 8.0 or IBM SPSS 25 and a *p* value < 0.05 was considered as statistically significant. Survival was analyzed using Kaplan–Meier method and log-rank test, followed by calculation of mortality HRs with 95% confidence intervals (CI) by Cox univariate and multivariate proportional hazard regression. T-test was used to compare the uptake (preclinical PET) between HAL-treated and Control mice and in comparing clinical parameters based on PET grouping. Significance of differential gene expression was determined by using DESeq2, where raw read counts are modeled using a negative bionomial distribution, following library size normalization and gene-wise dispersions estimation. Mice batch was included as a co-factor in the model to account for inter-batch variation. Hypothesis testing was performed using the Wald-test following between the HAL-treated and Control groups. Benjamini–Hochberg procedure used to calculate adjusted P values to correct for multiple hypothesis testing.

## Results

### Hepatic artery ligation induced radiotracer changes in xenografts

**Fig. 1:**
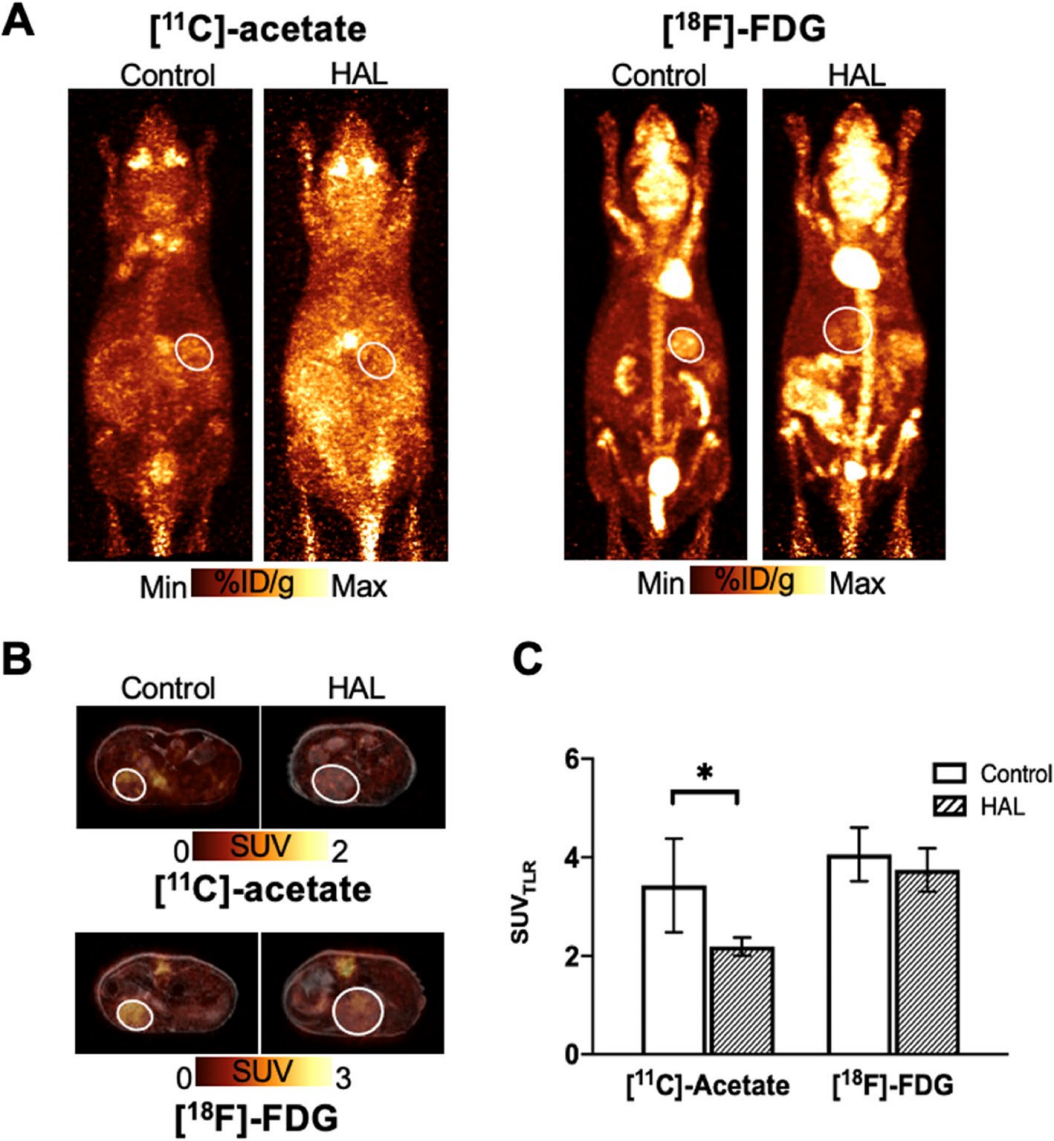
Dual-tracer [^11^C]-acetate and [^18^F]-FDG PET-MR imaging of HAL-treated and Control mice. Each mouse underwent sequential PET-MR imaging after intravenous injection of [^11^C]-acetate then [^18^F]-FDG. **A** Representative MIP PET and (**B**) co-registered transaxial PET-MR images of Control and HAL-treated mice injected with [^11^C]-acetate (**A**. left and **B**. top) and [^18^F]-FDG (**A**. right and **B**. bottom), respectively. Tumors were indicated by circle for clarity. **C** SUV_TLR_ of tumor [^11^C]-acetate and [.^18^F]-FDG uptake in HAL-treated and Control mice (*n* = 7 for Control, *n* = 5 for HAL-treated)

To investigate whether the PET phenotype identified in our local cohort was associated with underlying gene expression changes, dual-tracer PET-MR were performed on the orthotopic HCC mice. On MRI, tumor sizes between HAL-treated and Control mice were relatively similar at the time of imaging studies (HAL-treated vs Control, 133.6[122.4–383.2] vs 125.3[92.7–299.7] mm^3^, respectively, *p* = 0.43), indicating that HAL treatment did not significantly affect the tumor growth in vivo. No statistical difference was observed in the corresponding [^18^F]-FDG uptake (HAL-treated vs Controls, SUV_TLR_ 3.73[3.12–4.35] vs 3.86[3.7–5.29], *p* = 0.83) while [^11^C]-acetate uptake was significantly reduced in the HAL-treated mice when compared to the Control mice (SUV_TLR_ 2.14[2.05–2.21] vs 3.11[2.75–5.43], *p* = 0.02, respectively) (Fig. 1A-C). These results indicate that [^11^C]-acetate uptake could be a useful biomarker for imaging tumor hypoxia in vivo*.*

### Hypoxia-induced gene expression signature

**Fig. 2:**
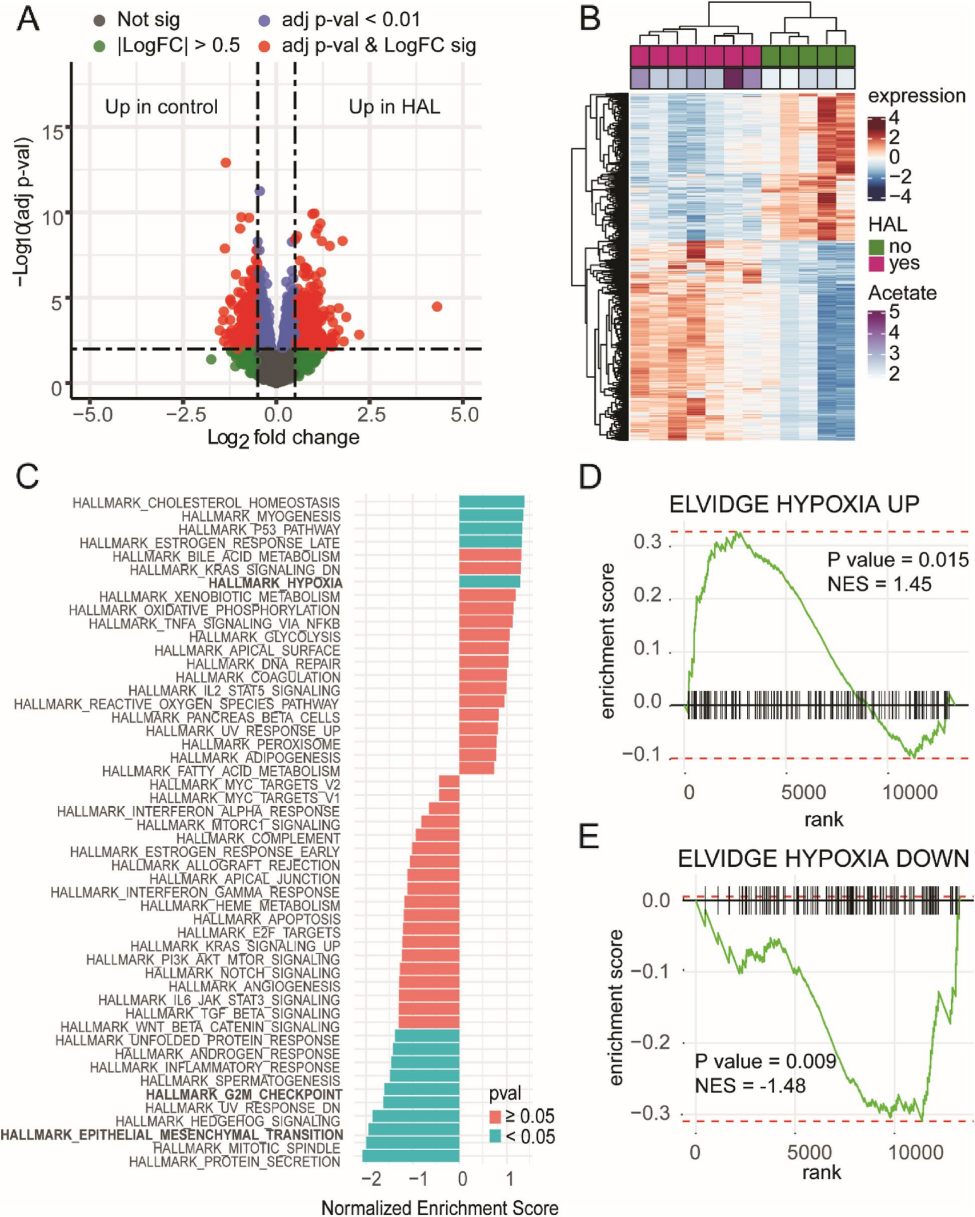
(**A**) Volcano plot, (**B**) Heat map and (**C**) Gene expression signatures reflect hypoxic state of HAL-treated HCC xenografts. (**A**) Differential gene expression analysis of Control versus HAL-treated HCC xenografts, (**D**) GSEA analysis showing enrichment of MSigDB Hallmark pathways in HAL-treated versus Control samples. GSEA plot of genes up (**D**) and down (**E**) regulated during hypoxia from Elvidge et al.

Based on the differential expression analysis of genes derived from the tumor sample between the HAL-treated and Control groups, we identified 232 upregulated and 143 down-regulated genes in the HAL-treated versus Control groups (Fig. 2A, Table S1, adjusted *p*-val < 0.01, |logFC|> 0.5). These genes segregated the HAL-treated and Control samples into two distinct clusters based on hierarchical clustering (Fig. 2B).

To assess the pathways that are perturbed in the HAL-treated versus Control groups, gene set enrichment analysis (GSEA) was performed against MSigDB HALLMARK signatures (Fig. 2C). A number of pathways were found to be significantly up or downregulated in HAL-treated compared with the Control samples. As expected, the hallmark hypoxia signature was significantly upregulated in the HAL-treated group. On the other hand, Hallmark G2M checkpoint and Hallmark epithelial-mesenchymal transition (EMT) were found to be significantly downregulated which is consistent with the knowledge that hypoxia can induce cell cycle arrest and epithelial-mesenchymal transition [20, 21].

To further validate that the HAL-treated model is indeed representative of hypoxic conditions at the molecular level, we selected hypoxia up and downregulated gene signatures from Elvidge et al. [22]. As expected, genes that upregulated hypoxia signature are significantly positively enriched in the HAL-treated versus the Control groups (Fig. 2D, NES = 1.45, *p* = 0.015), while the downregulated hypoxia signature is significantly negatively enriched in HAL-treated versus the Control groups (Fig. 2E, NES = -1.48, *p* < 0.01).

### [^11^C]-acetate uptake-associated gene signature on TCGA cohort

**Fig. 3:**
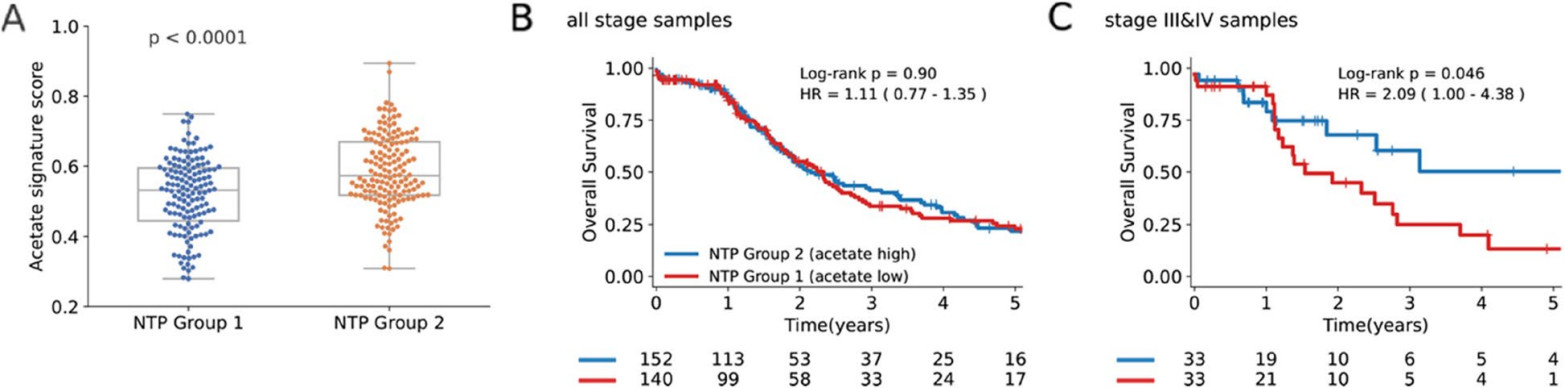
[^11^C]-acetate uptake gene signature is prognostic in late stage TCGA HCC patients. (**A)** GSVA score of genes upregulated in Control versus HAL-treated samples applied to TCGA HCC samples and grouped based on NTP prediction. *P*-value is calculated using Wilcoxon signed-rank test between the two groups. (**B**) Kaplan–Meier estimate of NTP group 1 (median OS = 2.22 years) versus group 2 (median OS = 2.23 years) using all TCGA HCC samples (*n* = 295). (**C)** Kaplan–Meier estimate of NTP group 1 (median OS = 1.67 years) versus group 2 (median OS = 2.05 years) using only stage III & IV TCGA HCC samples (*n* = 66)

Having demonstrated that HAL-treated xenograft is a robust model of hypoxia and with reduced [^11^C]-acetate uptake observed in HCC at the molecular level, we sought to use the gene expression data to create a signature representative of [^11^C]-acetate uptake. A total of 82 differentially expressed genes between the HAL-treated and Control groups was used to classify TCGA samples into one of two groups based on the Nearest Template Prediction (NTP) algorithm (Methods, Table S 2). In order to determine which NTP group is associated with high or low [^11^C]-acetate uptake based on the HAL-treated xenograft models, we used Gene Set Variation Analysis (GSVA) to calculate an acetate signature score using the upregulated genes in the Control group for each TCGA-LIHC sample (Fig. 3A) [18]. Samples in NTP group 1 has significantly lower acetate signature score compared with NTP group 2 (*p* < 0.001), thus samples in NTP group 1 can be considered to be low [^11^C]-acetate uptake, while NTP group 2 can be considered to have high [^11^C]-acetate uptake.

With the TCGA-LIHC samples classified into the NTP groups, a survival analysis was performed to compare the difference in OS between the two groups. When using all TCGA-LIHC samples, NTP group 1 and 2 had similar outcomes with median OS 2.23 vs 2.22 years (*p* = 0.90, HR 1.11 [95%CI 0.77–1.35]). However, when survival analysis was applied to late-stage patients, NTP group 1 had significantly worse survival with median OS 1.67 vs 2.05 years (*p* < 0.046, HR 2.09 [95%CI 1.00–4.38]) (Fig. 3B&C).

### PET phenotype on local cohort

To investigate the prognostic ability of tumor [^11^C]-acetate uptake, and by inference its correlation with hypoxia gene signature, we retrospectively analyzed HCC patients that had undergone dual-tracer PET for staging in our institute (Fig. S1). Diagnosis of HCC was either based on histological confirmation or by composite clinical reference standards [23]. As HCC is highly heterogenous, the radiotracer uptake patterns were determined by the predominate appearance of the tumor. Small foci of intra-tumoral radiotracers uptake were not considered. In patients with multifocal disease, the radiotracer uptake of the largest intrahepatic lesion was used for analysis. Of the 197 HCC patients included for analysis, 162 (82.23%) had [^11^C]-acetate avid, 125 (63.45%) [^18^F]-FDG avid, and 102 (51.78%) both [^18^F]-FDG and [^11^C]-acetate avid. The mean age of the cohort was 61, 160 (81.22%) were male with a mean size HCC tumor size of 5.79 cm (95%CI 5.03–6.55 cm). The mean follow-up of the cohort was 653 days, and a total of 83 (42.13%) patients died at the time of the study.

**Fig. 4:**
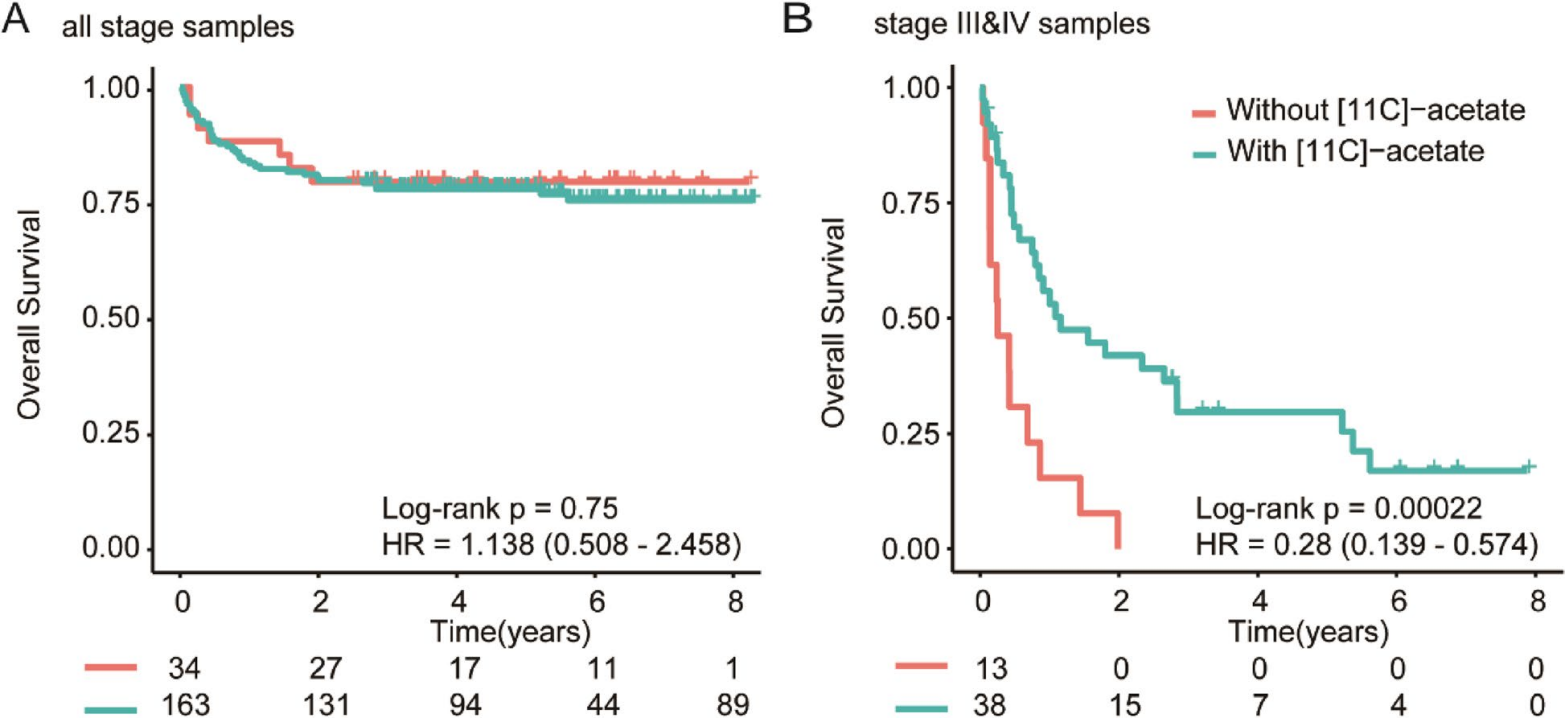
[^11^C]-acetate uptake is prognostic in late-stage HCC patients. **A** Kaplan–Meier estimate of all HCC patients undergoing staging with dual-tracer PET-CT (*n* = 197). Overall survival (OS) is similar between HCC with and without [^11^C]-acetate uptake. (**B**) Kaplan–Meier estimate of stage III & IV HCC tumors (*n* = 51) with and without [^11^C]-acetate uptake showing poorer OS in tumors without [^11^C]-acetate uptake

No statistical difference in OS was seen in patients with HCC with and without [^11^C]-acetate uptake with median OS 2.18 vs 2.00 years (*p* = 0.75, HR 1.138 [95%CI 0.77–2.46]). When applied to the 51 (27.41%) late-stage HCC patients, tumors without [^11^C]-acetate uptake had significantly worst OS compared with those with [^11^C]-acetate uptake, mean OS 0.25 versus 1.21 years (*p* < 0.0001, HR 0.28 [95%CI 0.14–0.57]) (Fig. 4). Patient characteristics and PET-CT images (Figs. S2 and 3) of the patients with late-stage disease are summarized in Table 1. Multivariable analysis of late stage HCC patients showed that advanced age (> 60 years), diffuse disease and presence of portal vein thrombus were associated with poor prognosis while tumor [^11^C]-acetate uptake was the most significant independent predictor for OS in late stage HCC patients in our cohort (Table 2).

**Table 1:** Characteristics of late stage (stage III & IV) Hepatocellular Carcinoma (HCC) patients undergoing dual-tracer PET-CT for staging

	Overall	[^11^C]- acetate avid HCC	[^11^C]-acetate non-avid HCC (%)	*P*
No of Patients	51	38	13	
Median age (years)	60.1 (± 14.4)	61.8	59.1	0.57
Gender (% male)	44 (86.3%)	33 (86.8%)	11 (84.6%)	> 0.99
Serum AFP (ng/ml)	30,351.6 (± 89,763.5)	29,779.4 (± 88,934.9)	30,353.0(± 89,763.2)	0.04
Aetiology				
Hepatitis B	34 (66.7%)	24 (63.2%)	10 (76.9%)	0.50
Hepatitis C	3 (5.9%)	3 (7.9%)	0 (0.0%)	0.56
Multiple etiologies	18 (35.3%)	13 (34.2)	5 (38.5)	> 0.99
Tumor features				
HCC staging III/IV (%)	33 (64.7%)	25 (65.8%)	8 (61.5%)	> 0.99
Tumor size (cm)	10.4 (± 5.49)	9.0	14.4	0.12
Focal disease (%)	29 (56.9%)	23 (60.5%)	6 (46.2%)	0.52
PV thrombosis (%)	39 (76.5%)	29 (76.3%)	10 (76.9%)	> 0.99
[^18^F]-FDG avid (%)	40 (78.4%)	27 (71.1%)	13 (100%)	0.046
Treatment				
Resection (%)	12 (23.5%)	9 (23.7%)	3 (23.1)	> 0.99
TACE (%)	11 (21.6%)	7 (18.4)	4 (30.8)	0.70
SIRT (%)	5 (9.8%)	5 (13.2)	0 (0)	0.31
Systemic therapy (%)	5 (9.8%)	5 (13.2)	0 (0)	0.31

**Table 2:** Univariate and multivariate proportional hazards regression analysis in predicting overall survival (OS) in late stage (stage III & IV) Hepatocellular Carcinoma (HCC) patients undergoing dual-tracer PET-CT for staging. Shaded boxes denote statistically significant variables

	**Univariate analysis**		**Multivariate analysis**	
	**HR (95% CI)**	***P***	**HR (95% CI)**	***P***
Age > 60 years	1.00 (1–1)	0.08	2.70 (1.30–5.70)	0.007
Gender (male)	0.35 (0.15–0.82)	0.016	0.5 (0.20–1.26)	0.143
Solitary	0.95 (0.51–1.80)	0.88	0.4 (0.19–0.91)	0.028
Tumor Size > 4 cm	1.70 (0.62–14.90)	0.3	0.67 (0.17–2.6)	0.57
PV thrombosis	1.60 (0.77–3.5)	0.20	2.8 (1.05–7.57)	0.041
AFP > 200 ng/ml	1.90 (1–30.5.00)	0.051	0.86 (0.38–1.96)	0.721
With [^11^C]-acetate	0.28 (0.14–0.57)	0.00048	0.15 (0.06–0.42)	0.00043
With [^18^F]-FDG	2.20 (1.00–4.80)	0.051	1.94 (0.63–5.96)	0.248
Without aetiology	0.69 (0.36–1.30)	0.26	0.58 (0.30–1.20)	0.137

## Discussion

In this study, we have demonstrated through orthotopic HCC xenografts that reduction in [^11^C]-acetate HCC uptake is associated hypoxia, and we developed a gene expression signature for [^11^C]-acetate uptake. When applied to the TCGA-LIHC dataset, advanced HCC patients with the lower acetate signature score have poorer overall survival (OS) and in our local cohort, [^11^C]-acetate uptake is also an independent predictor for OS in advanced HCC patients [24].

PET has long been a non-invasive imaging modality to probe cellular metabolism, and uptake of [^18^F]-FDG, the most commonly used radiotracer, has been shown to correlate with many molecular changes in cancers, including the expression of hypoxic gene signatures [25]. However, the clinical utility of [^18^F]-FDG in predicting tumor hypoxia in HCC is limited due to its poor sensitivity. With HCC cells relying on other carbon sources, most notably acetate, the addition of [^11^C]-acetate as a secondary radiotracer significantly improved the overall sensitivity of PET in detecting HCC, from around 50% to over 90% [6, 8, 9]. Therefore, dual-tracer PET is necessary to objectively investigate the relationship between hypoxia-induced gene expression and radiotracer uptake in HCC.

It was noteworthy that there is a discrepancy between the clinical observations and in vitro cell culture findings. While in vitro studies have confirmed increased acetate uptake in multiple types of cancers under hypoxia, [^11^C]-acetate was taken up preferentially in well differentiated and [^18^F]-FDG in poorly differentiated HCCs [6, 26]. Indeed, the expression of glucose transporters was significantly higher in hypoxic tumors, which tend to have higher [^18^F]-FDG uptake [27]. We speculate the contradictory observations seen in our preclinical studies could be the result of the intrinsic property of [^11^C]-acetate PET rather than a fundamental difference in tumor metabolism in HCC [10, 28]. Increased [^11^C]-acetate uptake is often observed under hyper-vascular environment [29]. Thus, [^11^C]-acetate uptake in vivo is not a true reflection of overall exogenous acetate uptake as seen in cell line studies but rather tumor perfusion. Indeed, when [^11^C]-acetate PET was initially employed, it was used for measuring myocardial perfusion where the lack of [^11^C]-acetate uptake corresponds to areas of cardiac hypoperfusion and hypoxia [30].

While the exact mechanism of [^11^C]-acetate in HCC remains elusive, the association between this phenotypic changes and hypoxia gene signature has significant clinical implications. Heterogeneity in tumor microenvironment is well-known in contributing to radiation and chemotherapy resistance, as well as a driver of more aggressive behavior, metastases, and poorer overall outcomes. Analysis using data from TCGA-LIHC confirmed that the gene expression changes secondary to hypoxia as simulated by HAL are associated with poorer prognosis in advanced-stage HCC. Combined with our local cohort that also showed late stage patients with non-[^11^C]-acetate avid HCC also had poorer survival, this implies that [^11^C]-acetate uptake can be a biomarker to identify patients or lesions that expressed this hypoxia gene signature, and therefore more likely to be aggressive and resistant to treatment [31].

There are several limitations to the study. First, only one cell line was investigated, and hence could not account for all the different genetic profiles and etiology backgrounds of this disease given the heterogeneity of this disease. However, we have demonstrated our findings in a human cohort (both local and the public database) and multivariant analysis has identified other well recognized predictors of survival in these patients such as age, diffuse disease and portal vein thrombus, implying our results are generalizable [32]. Second, even though hypoxia is induced through HAL, the degree of hypoxia in each tumor may be different and was not quantified. Although not routinely used in clinical practice, inclusion of novel hypoxia radiotracers such as [^18^F]-FAZA (^18^F-fluoroazomycin-arabinozide) in the future could allow us to identify a more specific and accurate biomarker [33]. Third, our animal study results are only applicable in late-stage HCC patients in both the TCGA and local cohorts. One potential explanation could be the treatment differences between early and late-stage diseases. In early stage HCC, curative treatments are favored compared with locoregional or systemic therapies for late-stage [34]. As resection and/or transplant are not affected by underlying genomic changes, the outcomes between different cohorts may not be significant. Whereas in late-stage disease, chemo- and/or radio-resistance often lead to disease progression and patient demise, significant difference in outcomes between those with and without acetate uptake gene signature is anticipated. Future prospective studies that capture both genomic and radiological data is necessary to conclusively validate our findings.

## Conclusions

In summary, we have shown that hypoxia in HCC induced by HAL resulted in a significant reduction in [^11^C]-acetate but not [^18^F]-FDG uptake using orthotopic HCC mice model. RNA-sequencing analyses showed that this metabolic change is associated with the expression of tumor hypoxia-related genes. Furthermore, upregulation of hypoxia gene signature is correlated with a poor prognosis in patients with advanced HCC. Collectively our findings demonstrate the potential of [^11^C]-acetate PET as a predictive biomarker for tumor hypoxia in HCC.

### Supplementary Information

**Supplementary Material 1**.

**Supplementary Material 2**.

## Data Availability

Not applicable.

## Abbreviations

[^18^F]-FAZA [^18^F]: Fluoroazomycin Arabinoside
[^18^F]-FDG [^18^F]: Fluorodeoxyglucose
HAL: Hepatic Artery Ligation
HCC: Hepatocellular Carcinoma
OS: Overall Survival
PET-CT: Position Emission Tomography - Computed Tomography
PET-MR: Position Emission Tomography - Magnetic Resonance
RNA: Ribonucleic Acid
SUV: Standardized Uptake Value
TCGA: The Cancer Genome Atlas Program
VOI: Volume of Interest
