# Supplementary material for: Prognostic PET [11C]-acetate uptake is associated with hypoxia gene expression in patients with late-stage hepatocellular carcinoma – a bench to bed study
Source: Cancer Imaging. 2024 Mar 22;24:42. doi: 10.1186/s40644-024-00685-9 (PMC10958914; doi:10.1186/s40644-024-00685-9)
Supplement: Supplementary file 1 — Supplementary Material 1. [file 40644_2024_685_MOESM1_ESM.docx]

**SUPPLEMENTAL MATERIAL**

**Creation of** **Orthotopic HCC Xenografts**

**Cell culture**

Human hepatocellular carcinoma cell line MHCC97L (from the Liver Cancer Institute, Fudan University, Shanghai, China; (1) were maintained in Dulbecco’s Modified Eagle’s Minimal Essential Medium (DMEM) with high glucose (Gibco, Thermo Fisher Scientific, Waltham, MA, USA) supplemented with 10% foetal bovine serum (FBS), penicillin at 50 units/mL, streptomycin at 50 µg/mL and sodium bicarbonate at 3.7 g/L under aseptic conditions at 37 °C in a humidified incubator at 5% CO_2_. Cells were regularly tested to be negative for *Mycoplasma*, and their identity was validated by short tandem repeat profiling. (2)

**Subcutaneous injection, orthotopic liver implantation and hepatic artery ligation**

All animal procedures were performed in accordance with the Committee on the Use of Live Animals in Teaching and Research (CULATR) in the Centre for Comparative Medicine Research (CCMR) at the University of Hong Kong, a program accredited by the Association for the Assessment and Accreditation of Laboratory Animal Care International (AAALAC). The animals used in the study were female BALB/cAnN-nu (Nude) mice at the age of 6–8 weeks, weighted at 20 g ± 2 g. Mice were housed in individual ventilated cages (IVC), up to 5 per cage, in an artificial day–night cycle facility. Food and water were provided ad libitum.

HCC xenografts creation and PET-MR imaging were performed using the previously optimized protocol (9). A suspension containing 5 × 10^6^ MHCC97L human HCC cells in serum-free medium and Matrigel (1:1, vol/vol; Corning Inc, Corning, NY, USA) was injected subcutaneously into the lower right flank of nude mice. Mice were monitored 2–3 times per week for health and well-being and signs of tumorigenesis. All mice developed subcutaneous tumor at the end of the study (4–6 weeks post inoculation). A total of 4 mice were used to create subcutaneous HCC xenografts.

Once the subcutaneous tumor reached 800 mm^3^ in volume as determined by caliper measurements, mice were culled, and each tumor was carefully removed, and subsequently cut into 1–2 mm^3^ size tumor for immediate implantation into the left liver lobe of the healthy nude mice to create orthotopic HCC xenografts. Mice were given buprenorphine subcutaneously (0.1 mg/kg) 30 minutes prior to the start of surgery. Mice were first anesthetized and laparotomy was performed with expose of the liver, followed by mechanical injury on the liver capsule using a needle for the insertion of tumor cube. Tumor cube was secured by suture. Mice were randomly divided into the HAL and control groups. Hepatic artery ligation was performed during orthotopic tumor implantation, by ligating the main branch of the hepatic artery using a fine thread under an operating microscope. Control group mice underwent the same orthotopic implantation but without HAL. The abdominal wall was closed by suture. Mice were monitored daily after surgery and supplied with multi-modal analgesia as per ethics approval of the CULATR. Mice were monitored 2–3 times per week for health and well-being and signs of tumorigenesis. All mice developed tumor in the liver prior to PET-MR imaging studies (approximately 4 weeks post inoculation), A total of 14 mice were created for this study, consisting of 7 HAL-treated and 7 sham-operated (Control) groups. 2 HAL mice were excluded from this study and culled at humane endpoint due to post-surgical complications, giving 5 HAL and 7 control mice (n=12) for imaging and RNA sequencing analyses. The sample sizes were selected taking into account the statistical considerations based on our published methodology and literature (9,10). Surgery and imaging procedures and data analyses were performed by two different researchers (KVT and XY) who are blinded to the mice allocation until the data have been analyzed. A third researcher (HY) who was aware of the allocation was only involved in animal care and did not participate in data collection and analyses.

**RNA sequencing and analyses of tumor samples**

After quality control procedures, Poly(A)^+^ mRNA library preparation and sequencing were performed at The University of Hong Kong (Centre for PanorOmic Sciences, Genomics and Bioinformatics Cores) using the KAPA stranded mRNA-Seq kit, to generate ~30 million 151 bp paired-end reads. As the transcriptomic data is derived from mouse xenografts, the RNA is a mixture of human tumor RNA and mouse stromal RNA. In order to resolve the species from which each read is derived, the disambiguate pipeline was used (3). Briefly, sequencing reads from each sample was first trimmed of adaptors using bbduk version 38.22 (4). Trimmed reads were then aligned separately against the human genome (hg19) and the mouse genome (mm10) using STAR version 2.7.2b (5). The disambiguate algorithm was then used to sort reads into those that uniquely align to either the human genome and the mouse genome. Ambiguous reads were discarded.

**Combined [^11^C]-acetate and [^18^F]-FDG (Dual-Tracer) PET-CT patient selection**

Consecutive adult patients who underwent dual-tracer PET/CT between January 2015 and December 2019 in our institution were retrospectively recruited from our department’s database. Patients included for analysis were treatment naïve patients with either radiological (based on either EASL or APASL guidelines or a LR-5 lesion using The Liver Imaging Reporting and Data System (LI-RADS) version 2018) or histological diagnosis of HCC prior to initiation of any treatment regimen. Conventional imaging (CT or MRI) must be performed within 3 months of dual-tracer PET-CT. In total, 197 patients fulfilled the inclusion criteria (Fig S1).

**Combined [^11^C]-acetate and [^18^F]-FDG (Dual-Tracer) PET-CT Scanning Protocol in human subjects**

All patients were fasted for at least 6 hours to achieve a blood glucose concentration <10 mmol/L before the injection of PET radiopharmaceuticals. [^11^C]-acetate (0.12 mCi/4.44 MBq per kilogram body weight) was administered through peripheral intravenous access, and limited whole-body imaging (from the base of the skull to the upper thighs) was performed 11 minutes after injection (6, 7). Fifteen minutes after the completion of [^11^C]-acetate imaging, [^18^F]-FDG was injected intravenously (0.078 mCi/2.88 MBq per kilogram body weight) and limited whole-body imaging was performed again 60 minutes after administration. Data acquisition was performed using an integrated in-line PET/CT scanner (GE Discovery 610 64-MSCT or GE Discovery MI 64-MSCT; GE Healthcare, Chicago, IL, USA) beginning with CT at 140 kV and 120–400 mA, a pitch of 0.984:1, and a tube rotation of 0.5 seconds. This was followed by PET with an emission acquisition time of 2 minutes per position for a 70 cm transverse field of view. Images were reconstructed with the standardized ordered-subset expectation maximization technique using 16 subsets and 2 iterations, with a 192×192 matrix for PET and a 512×512 matrix for CT. Reconstruction parameters were the same for both [^11^C]-acetate and [^18^F]-FDG PET (8).

**Combined [^11^C]-acetate and [^18^F]-FDG (Dual-Tracer) PET-CT image analysis in human cohort**

All dual-tracer PET/CT were reviewed in consensus between a board-certified radiologist and a clinical oncologist (KC and CC), both with >5 years of clinical experience in interpreting or reporting dual-tracer PET-CT. In all cases, patient history and findings on conventional imaging were taken into account when interpreting the images. A lesion was considered to be positive for either radiotracer on the basis of visual assessment of the degree of increased metabolism over background liver parenchyma, supported by calculation of the standardized uptake value (SUV) with a lesion-to-liver ratio of >1.20 and the uptake must be considered to be non-physiological without the context of being a benign lesion. A lesion was considered to be positive for dual-tracer PET/CT if it was hypermetabolic with either or both radiotracers. Lesions that were isometabolic or hypometabolic to background liver parenchyma were considered to be negative for the specific radiotracer (6).

**Supplementary Figures**


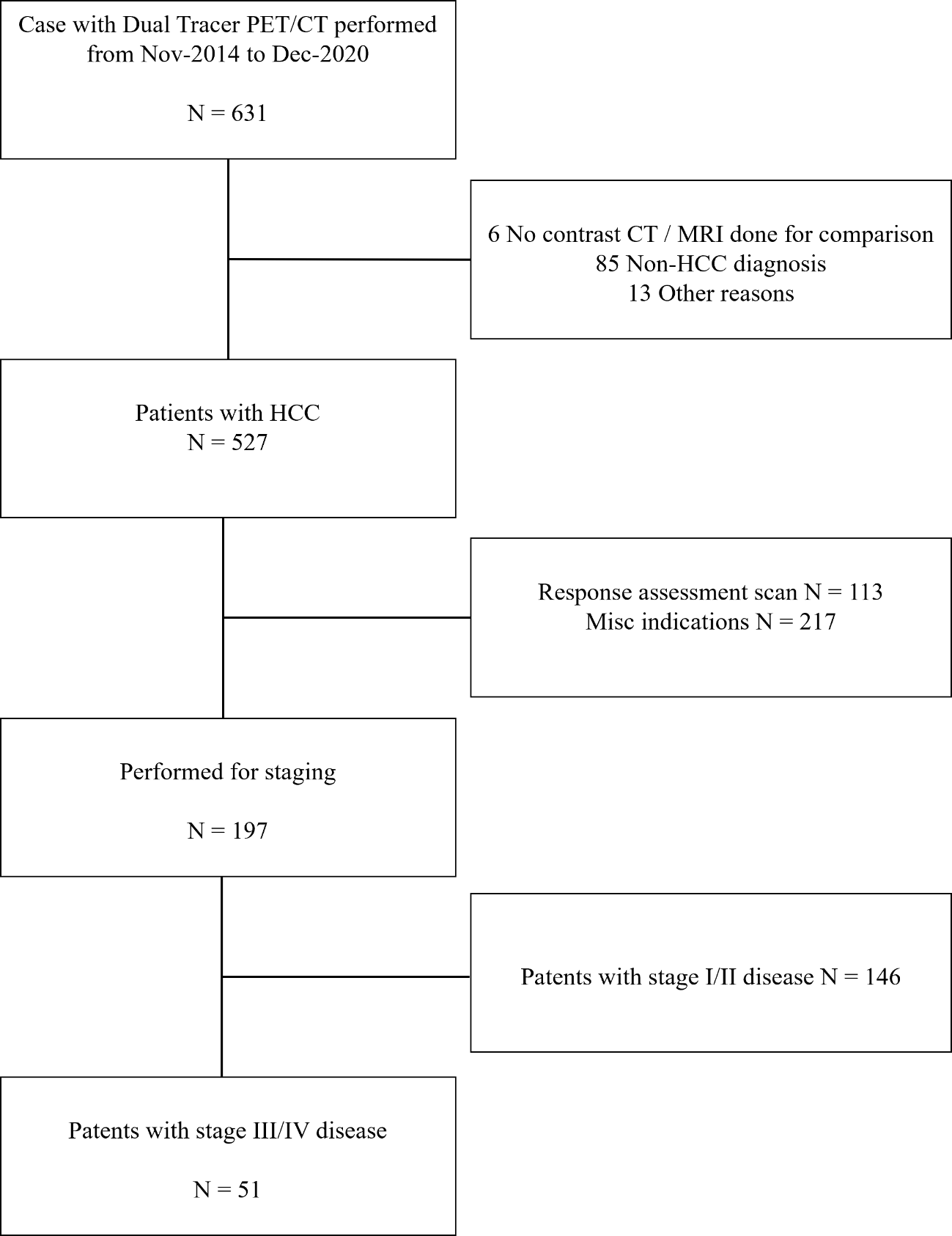


**Figure S1**. Flowchart of patient selection in local cohort.


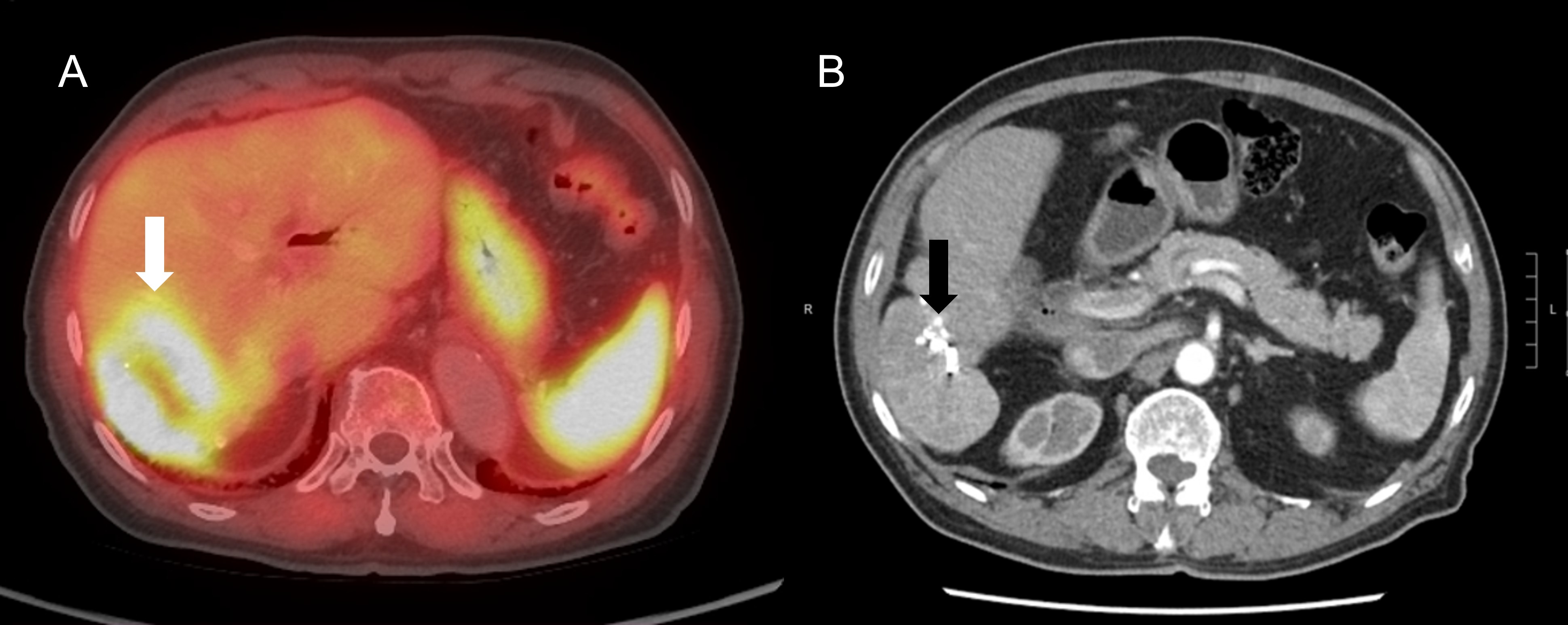


**Figure S2**. An 80 year-old man with chronic hepatitis B and a large HCC in the right lobe of the liver. (A) Axial PET/CT fused image of the HCC (white arrow) exhibiting high [^11^C]-acetate uptake. The patient underwent multiple locoregional treatments and remains alive after 65 months of follow-up. (B) Axial CT image of patient’s 3-year follow-up scan showing treatment response (black arrow).


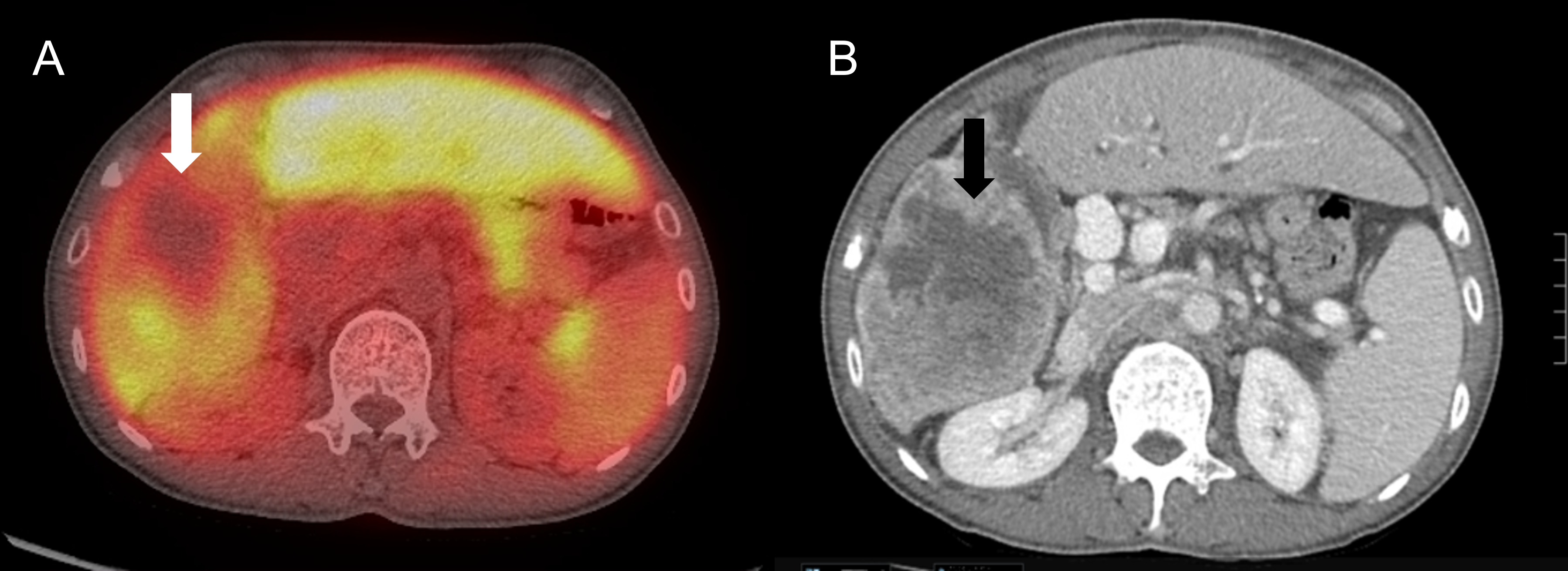


**Figure S3**. A 59 year-old man with chronic hepatitis B and multifocal HCC. (A) Axial PET/CT fused image of a non-avid [^11^C]-acetate tumour (red arrow). Despite locoregional and systemic treatments, the patient died 5 months later. (B) Axial CT image of patient’s last follow-up scan showing disease progression (yellow arrow).

**REFERENCES**

1. Li Y, Tang Z-Y, Ye S-L, Liu Y-K, Chen J, Xue Q, et al. Establishment of cell clones with different metastatic potential from the metastatic hepatocellular carcinoma cell line MHCC97. World Journal of Gastroenterology. 2001;7(5).

2. Tang Z-Y, Ye S-L, Liu Y-K, Qin L-X, Sun H-C, Ye Q-H, et al. A decade?s studies on metastasis of hepatocellular carcinoma. Journal of Cancer Research and Clinical Oncology. 2004;130(4):187-96.

3. Ahdesmäki MJ, Gray SR, Johnson JH, Lai Z. Disambiguate: An open-source application for disambiguating two species in next generation sequencing data from grafted samples. F1000Research. 2016;5.

4. Biggs PJ, Bushnell B, Rood J, Singer E. BBMerge – Accurate paired shotgun read merging via overlap. Plos One. 2017;12(10).

5. Dobin A, Davis CA, Schlesinger F, Drenkow J, Zaleski C, Jha S, et al. STAR: ultrafast universal RNA-seq aligner. Bioinformatics. 2013;29(1):15-21.

6. Ho CL, Yu SC, Yeung DW. 11C-acetate PET imaging in hepatocellular carcinoma and other liver masses. J Nucl Med. 2003;44(2):213-21.

7. Norenberg JP, Simpson NR, Dunn BB, Kiesewetter DO. Remote synthesis of [11C]acetate. International Journal of Radiation Applications and Instrumentation Part A Applied Radiation and Isotopes. 1992;43(7):943-5.

8. Lu R-C, She B, Gao W-T, Ji Y-H, Xu D-D, Wang Q-S, et al. Positron-emission tomography for hepatocellular carcinoma: Current status and future prospects. World Journal of Gastroenterology. 2019;25(32):4682-95.

9. Tan KV, Yang XX, Chan CY, Shi J, Chang H-C, et al. Non-invasive PET/MR Imaging in an Orthotopic Mouse Model of Hepatocellular Carcinoma. Journal of Visualized Experiments. 2022(186). e63958, doi:10.3791/63958.

10. Vanhjove C, Bankstahl JP, Kramer SD, Visser E, Belcari N, et al. Accurate molecular imaging of small animals taking into account animal models, handling, anaesthesia, quality control and imaging stystem performance. EJNMMI Physics. 2015;2(31):1-25.
